# A Novel Genome-Wide Association Study Approach Using Genotyping by Exome Sequencing Leads to the Identification of a Primary Open Angle Glaucoma Associated Inversion Disrupting *ADAMTS17*

**DOI:** 10.1371/journal.pone.0143546

**Published:** 2015-12-18

**Authors:** Oliver P. Forman, Louise Pettitt, András M. Komáromy, Peter Bedford, Cathryn Mellersh

**Affiliations:** 1 Kennel Club Genetics Centre, Animal Health Trust, Newmarket Suffolk, CB8 7UU, United Kingdom; 2 Department of Clinical Science & Services, Royal Veterinary College, University of London, Hawkshead Lane, Hatfield, Hertfordshire, AL9 7TA, United Kingdom; 3 Department of Small Animal Clinical Sciences, College of Veterinary Medicine, Michigan State University, Veterinary Medical Center, 736 Wilson Road, East Lansing, MI, 48824–1314, United States of America; University of Alabama at Birmingham, UNITED STATES

## Abstract

Closed breeding populations in the dog in conjunction with advances in gene mapping and sequencing techniques facilitate mapping of autosomal recessive diseases and identification of novel disease-causing variants, often using unorthodox experimental designs. In our investigation we demonstrate successful mapping of the locus for primary open angle glaucoma in the Petit Basset Griffon Vendéen dog breed with 12 cases and 12 controls, using a novel genotyping by exome sequencing approach. The resulting genome-wide association signal was followed up by genome sequencing of an individual case, leading to the identification of an inversion with a breakpoint disrupting the *ADAMTS17* gene. Genotyping of additional controls and expression analysis provide strong evidence that the inversion is disease causing. Evidence of cryptic splicing resulting in novel exon transcription as a consequence of the inversion in *ADAMTS17* is identified through RNAseq experiments. This investigation demonstrates how a novel genotyping by exome sequencing approach can be used to map an autosomal recessive disorder in the dog, with the use of genome sequencing to facilitate identification of a disease-associated variant.

## Introduction

It is well documented that population structure in the purebred dog can help to facilitate genome-wide association study (GWAS) approaches [1]. The development of most modern breeds within the last 200 years from small numbers of founding individuals has led to high levels of linkage disequilibrium (LD) within breeds. These high levels of LD lead to very strong signals of association being produced from GWASs for autosomal recessive diseases, even with very modest sample numbers [2]. Closed breeding populations, high levels of inbreeding and the extensive use of popular sires (dogs that closely fit the standard for a particular breed) can lead to rapidly emerging autosomal recessive disorders, as rare deleterious alleles are rapidly amplified. An example of an emerging autosomal recessive disorder is primary open angle glaucoma (POAG) in the Petit Basset Griffon Vendéen (PBGV).

**Fig 1:**
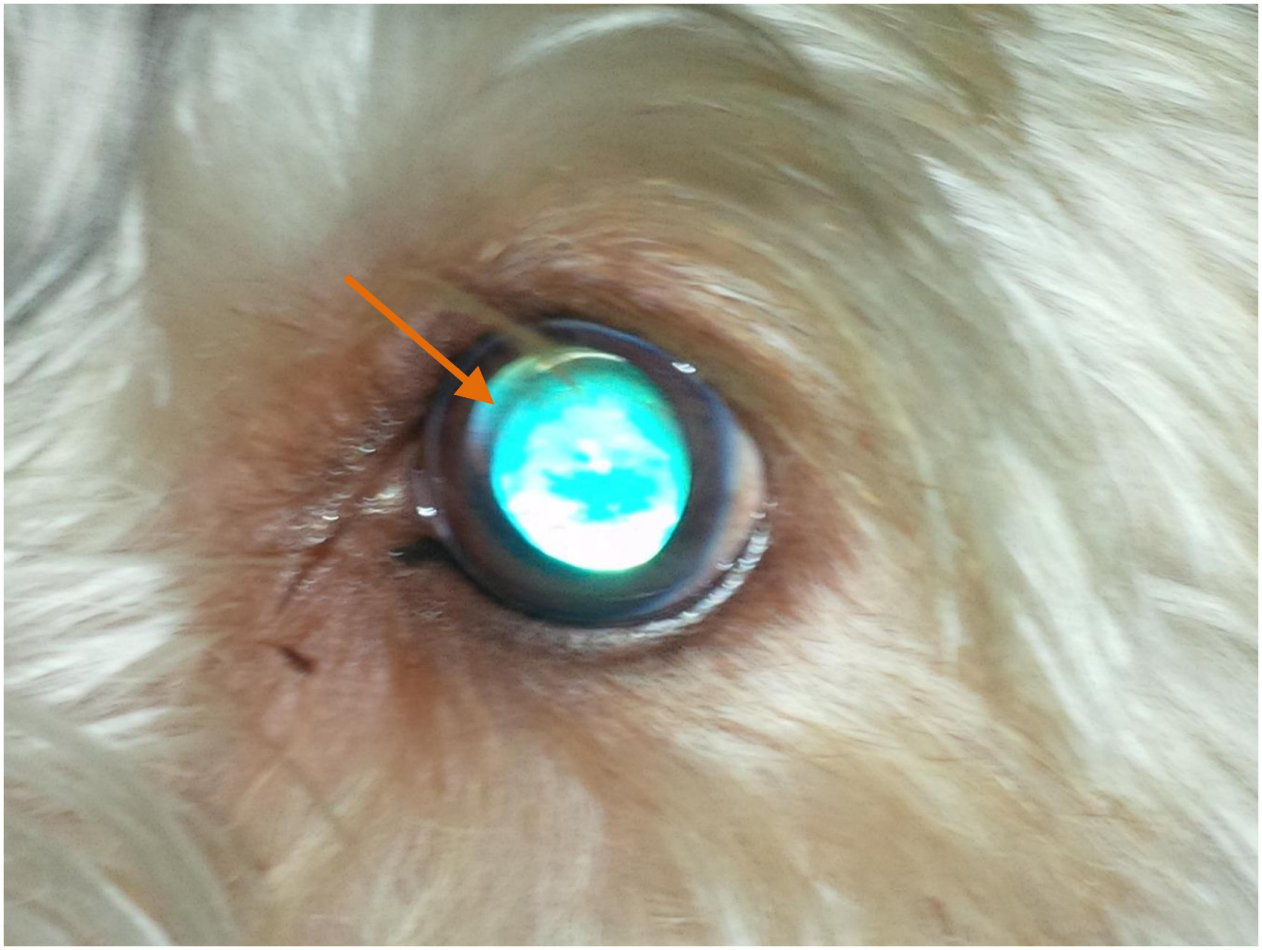
POAG case eye image. Left eye, 4 year old male PBGV: The eye is normotensive (18 mm. Hg.), but an aphakic crescent indicating lens subluxation is visible within the dorsal part of the dilated pupillary aperture.

The first recognised case of POAG in the PBGV was identified in the United Kingdom in 1996 and recent survey work completed in 2014 has demonstrated a 10.4% prevalence for the disease (personal communication, Peter Bedford). The initial clinical features of POAG are usually seen in 3 to 4 year old dogs of either sex, the disease being characterised by a small, sustained rise in intraocular pressure (IOP) and lens subluxation. In approximately one third of affected dogs phacodonesis and the appearance of the aphakic crescent associated with lens subluxation are seen before a noticeable rise in IOP (Fig 1). There is no pectinate ligament abnormality and the iridocorneal angle remains open until the late stages of the disease, when globe enlargement has developed. Retinal degeneration and a cupping deformation of the optic papilla are only seen in late disease. Pain is not a feature and the quiet, chronic clinical nature of this disease means that often owners only become aware of the presence of POAG when either the globe enlargement or a vision problem becomes noticeable.

As POAG is an autosomal recessively inherited disease, mapping of which are facilitated by the high levels of LD described, we designed a novel GWAS approach using genotyping by exome sequencing methodology with 12 cases and 12 controls with the dual aim of identifying both the disease-associated locus and causal variant for POAG through a single experiment.

## Results

### Genome-wide association study by exome sequencing (POAG)

Exome sequencing was carried out using a commercially available human exome capture kit to capture the exomes of 12 POAG cases and 12 breed matched control dogs. Illumina sequencing produced a 15.0 Gb dataset of 250 bp paired-end reads (sufficient for low coverage of ~5x). Alignment to the canine reference sequence CanFam3.1 and variant calling across all 24 individuals identified a total of 841,115 SNP and indel calls (variants). After filtering variants with a minor allele frequency (MAF) of less than 5% and genotyping frequency (GF) of less than 80%, 61,977 remained.

**Fig 2:**
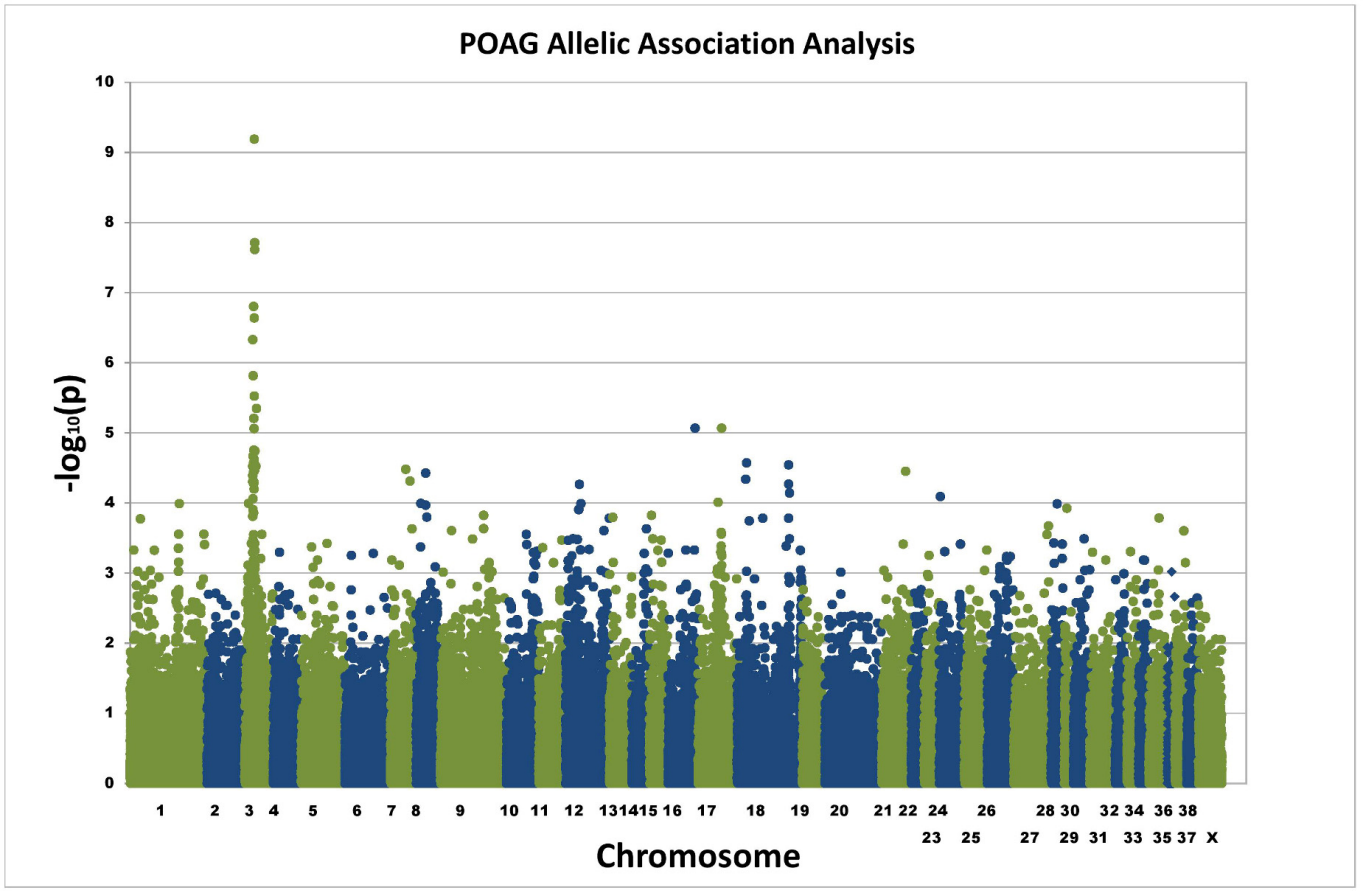
Allelic association plot for POAG GWAS. Exome sequencing was used to generate SNPs for 12 POAG cases and 12 controls. Allelic association analysis identified a single signal on chromosome 3 of genome-wide significance.

Basic allele association analysis identified a single signal of genome-wide significance on canine chromosome 3 (p_raw_ = 6.15x10^-10^) (Fig 2). The genomic inflation factor (based on median chi-squared) was 1.34. Correction for the effects of population substructure was performed using a mixed model approach (EMMAX) [3] and the strong single signal on chromosome 3 remained (p = 1.34x10^-9^) (S1 Fig). The adjusted genomic inflation factor (based on median chi-squared) was 1.04.

**Fig 3:**
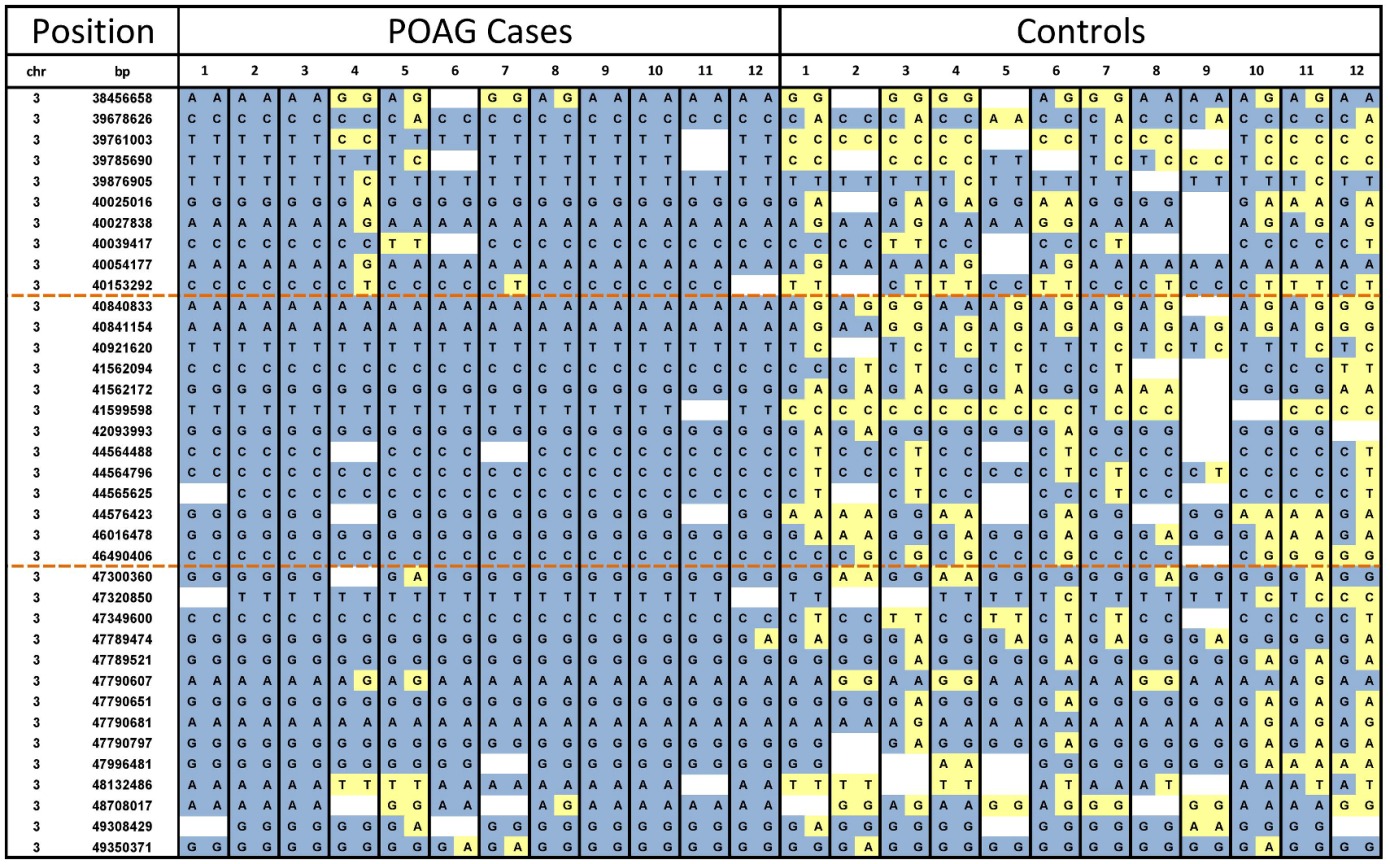
Genotyping data across the POAG disease-associated interval. Visualisation of the genotyping dataset across chromosome 3 was used to identify the disease-associated interval. Loss of homozygosity in cases defined the boundaries of the associated interval (orange dashed lines). Minor alleles are shown in yellow and major alleles in blue.

Visual analysis of the raw genotyping data revealed a disease associated interval of chr3:40,153,292–47,300,360 based on the CanFam3.1 genome build (Fig 3). All cases were homozygous for the disease-associated haplotype. The disease-associated interval contained 28 genes, including *ADAMTS17*, a potential glaucoma candidate gene. A list of interval genes can be found in S1 Table. As all cases were homozygous for the disease-associated haplotype the exome sequencing datasets were combined for all cases to increase read depth for interrogation of the disease-associated interval. As the human kit was used for target enrichment, capture of canine exons was incomplete (approximately 80%). For *ADAMTS17* additional exon resequencing was performed to cover all exons, in three POAG cases and three controls, although no coding or splice site variants were identified.

The SNP with the lowest p-value from the GWAS (top SNP) was a non-synonymous SNP in the *SYMN* gene (chr3:41,599,598). Conservation analysis across vertebrate species showed weak conservation of this residue, with a number of naturally occurring amino acids at this position. The variant is also predicted to be tolerated by SIFT. In total, 2,696 SNPs and indels were identified across the disease-associated interval, including 12 non-synonymous variants, although none segregated fully with disease status (i.e. homozygotes for the non-reference allele were present in both case and control sets). A list of non-synonymous variants with consequent predictions is shown in S2 Table.

**Fig 4:**
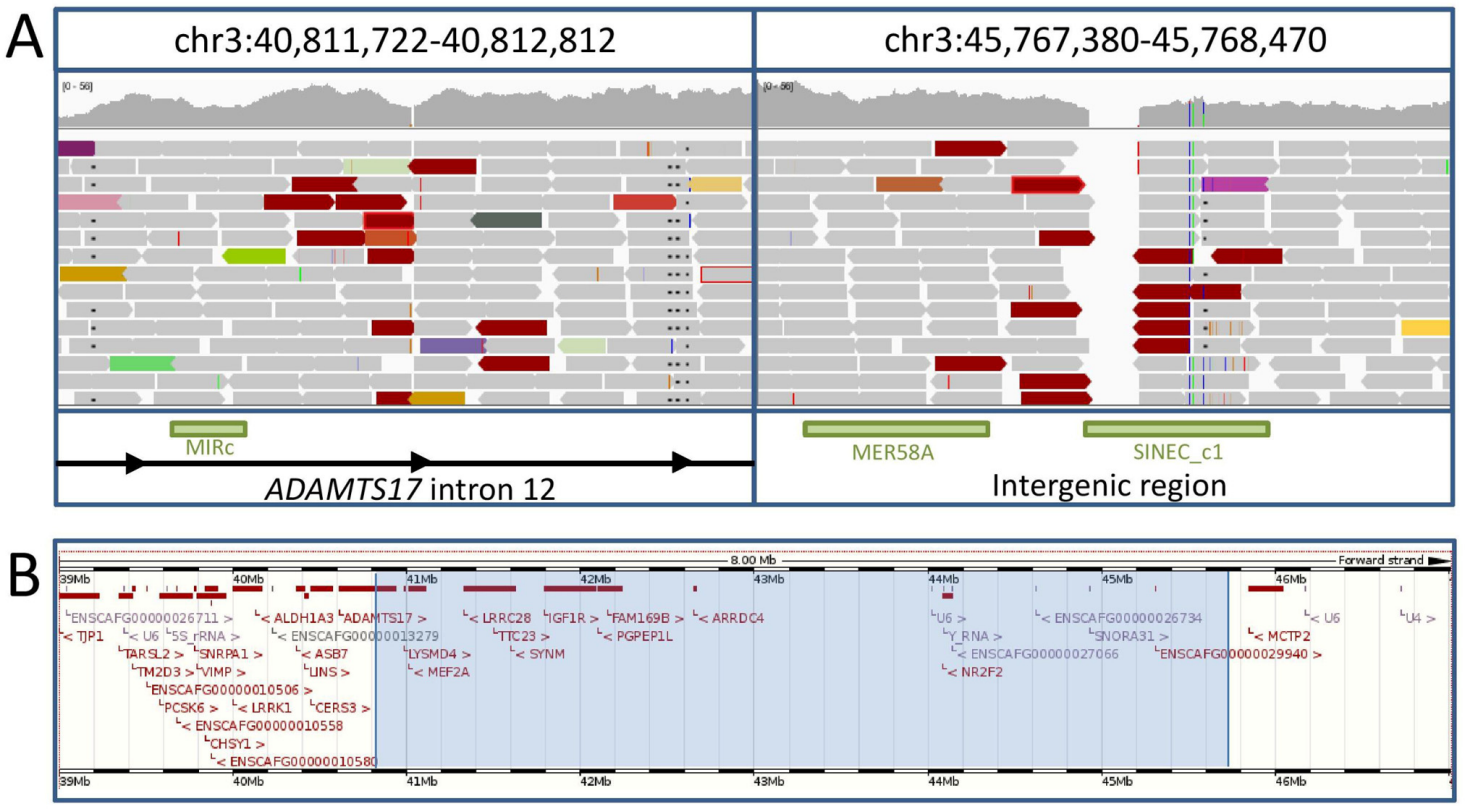
The POAG associated inversion. (A) Reads aligning across the inversion breakpoints. Red reads indicate a greater than expected insert size. Read mates for red reads align in the same direction, indicative of an inversion. There were five deleted bases at the 5’ inversion breakpoint and 78 deleted bases at the 3’ deletion breakpoint. Green boxes indicate repeat elements. (B) Overview of the genomic region covered by the inversion. The inverted region is highlighted in blue. Genotyping of the inversion was carried out to confirm the association with POAG. A total of 225 PBGVs were genotyped, including 28 POAG cases, of which 27 were homozygous for the inversion. Results are summarised in Table 1.

The disease-associated interval was further investigated by genome resequencing of a single POAG case. To consider intronic, exonic and intergenic regions in detail, sequence read alignments were visually scanned using the Integrative Genomics Viewer (IGV) [4]. Sequence read alignments indicative of a 4.96 Mb inversion were identified with breakpoints in intron 12 of *ADAMTS17* (chr3:40,812,274) and a downstream intergenic region (chr3:45,768,123) (Fig 4).

**Table 1:** Genotyping of an extended PBGV sample set for the POAG-associated inversion.

	+/+	INV/+	INV/INV
POAG cases	1	0	48
controls	92	71	0
**TOTALS**	**93**	**71**	**48**

Results of genotyping 212 PBGV for the POAG associated inversion, where + represents the reference allele and INV represents the inversion allele.

### Expression analysis

To gauge whether the inversion had an impact on gene expression, limited qRT-PCR experiments were performed. Tissues for RNA extraction were selected based on the availability of suitable case and control material and assessment of expression levels of *ADAMTS17* using RNAseq data generated in previous studies (data not shown). In a comparison of retinal cDNA from one POAG case against one control, results suggested a 2.4 fold increase in *ADAMTS17* expression upstream of the inversion for the POAG case relative to the control. No *ADAMTS17* expression was detected downstream of the inversion for the POAG case. (Full results are shown in S1 Dataset).

**Fig 5:**
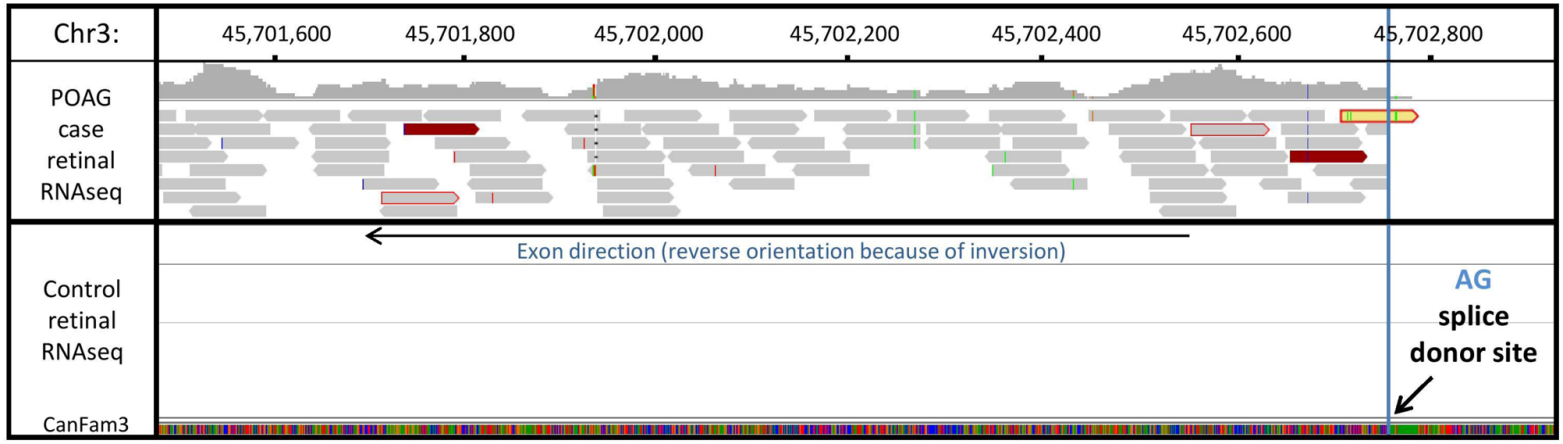
Example of novel exon formation through cryptic splicing. An example of a novel exon occurring due to a cryptic splicing event visualised through aligned RNAseq reads. A splice donor site and reads spanning to the previous exon (solid red) can be identified. There are no reads aligning to the region for the control individual (lower panel).

**Fig 6:**
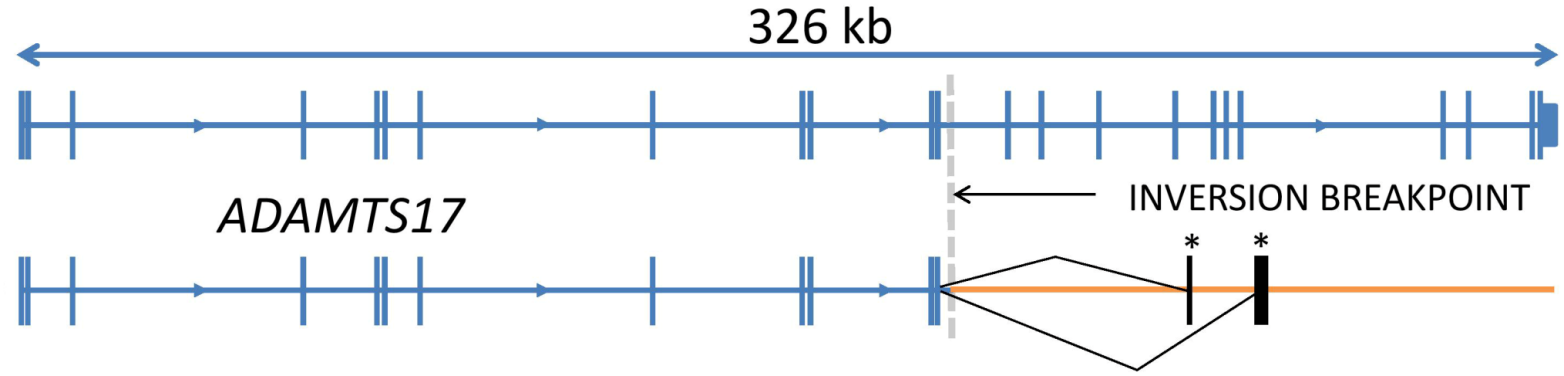
Schematic gene arrangements for the reference and inversion alleles. Transcript arrangements for the *ADAMTS17* reference gene and *ADAMTS17* after the inversion event. Novel exons are marked with an asterisk.

RNAseq data generated from retinal RNA of one POAG case, showed concordance with the results of qPCR analysis. Expression of novel exons as the result of cryptic splicing was observed after the final normally transcribed exon of *ADAMTS17* before disruption by the inversion. An example of a novel exon established through a cryptic splicing event is shown in Fig 5. A schematic diagram of *ADAMTS17* exon arrangement is shown in Fig 6.

Both of the sequences of the two independent novel exons contained stop codons after an aberrant sequence of amino acids (S1 File).

## Discussion

In this investigation we have demonstrated a novel GWAS approach using exome sequencing variants calls as the genotyping dataset. The major potential advantage of this approach is that a causal variant for a single gene disease could be identified in a single step, and would theoretically be the most strongly disease segregating variant with the lowest associated p-value (top SNP). Although the causal variant for POAG was not directly found by this method, due to the non-exonic location, a genome-wide significant locus was identified, enabling a disease-associated interval to be determined.

The causal mutation for POAG was eventually identified through a genome sequencing approach. Genome sequencing is an increasingly cost effective method of following up disease-associated intervals identified through GWAS. In contrast to other approaches such as exome sequencing and target enrichment, coverage is near to 100%, enabling identification of causal mutations in repetitive regions of the genome. Genome sequencing has recently been adopted to directly identify causal mutations in the dog [5–7] and is being used in human studies through projects such as the 100,000 genomes project, studying cancers and rare diseases [8]. However, structural variants still often present a challenge to smaller laboratories using genome sequencing as part of an investigation, making GWAS in many cases the most appropriate method of locus identification for single gene diseases, before using genome sequencing or other approaches to interrogate disease-associated intervals.

Although the *ADAMTS17* gene was majorly disrupted by the inversion, qPCR analysis on a limited number of samples showed little evidence of nonsense mediated decay, as expression of exons outside of the inverted region was not majorly affected. Analysis of RNAseq data revealed novel exon expression for *ADAMTS17* due to cryptic splicing occurring 3’ of the exons located immediately upstream of the inversion event. The unavailability of suitable antibodies targeting the 5’ region of *ADAMTS17* prevented western blot analysis to determine whether protein is still produced from the modified transcript sequence. Visual analysis of RNAseq data aligned to the genes flanking and within the inversion region suggested gene expression for these genes was not affected. We speculate that a mobile element (SINE) which spans the 3’ inversion breakpoint is likely to have been involved in the inversion mechanism, although there is no similar mobile element in the 5’ region. There is one previous report of a gene rearrangement involving *ADAMTS17* found in a patient with pregnancy-related acute promyelocytic leukemia [9]. This rearrangement resulted in a novel transcription product with the insertion of exon 15 of *ADAMTS17* between the PML and RARA genes. As the breakpoint within *ADAMTS17* identified in this study was within intron 12, it is unlikely that the mutation mechanism was shared between these two independent events.

Genotyping of an extended sample set was used to determine whether the identified inversion was fully concordant with POAG. Of the 212 PBGV assayed for the POAG associated inversion there was one discordant case. A single dog was reported as having clinical signs of POAG but was homozygous for the reference sequence. It could be speculated that POAG for this case is due to another clinical or genetic cause, or is a secondary form of glaucoma.

A Weill-Marchesani like syndrome (WMS) in humans was the first disease phenotype to be associated with mutations in the *ADAMTS17* gene. Clinical signs of WMS include glaucoma, ectopia lentis (lens luxation), lenticular myopia, spherophakia, and short stature [10]. It is interesting to speculate that as the PBGV is a miniature version of the Grand Basset Griffon Vendéen, artificial selection pressure for small size may have contributed to an increase in mutant allele frequency, assuming mutations in *ADAMTS17* could be contributing to small stature. An *ADAMTS17* splice donor site mutation has been associated with hereditary primary lens luxation in several breeds of dog [11, 12]. The range of phenotypes associated with *ADAMTS17* mutations suggests that the exact phenotypic presentation is dependent on the positioning and nature of the mutations within the *ADAMTS17* gene. The *ADAMTS17* gene is a member of the ADAMTS family of extracellular proteases [13]. Domains which are characteristic of the ADAMTS family include an N terminal protease domain and a C-terminal ancillary domain [14]. The inversion in intron 12 of *ADAMTS17* is within the proteolytic domain and is therefore likely to disrupt the enzymatic function of the protein. Phenotypic similarities between ADAMTS associated disease and Fibrillin1 (*FBN1*) associated disease, and functional evidence, suggest ADAMTS family members have a role in microfibril assembly and function [15]. It could be speculated that disruption of the maintenance of microfibrils in the eye are likely to lead to development of disease phenotypes such as lens luxation and glaucoma [16]. The role of the ADAMTS family members in glaucoma pathogenesis is further highlighted by the identification of a mutation in *ADAMTS10* as the cause of glaucoma in the Beagle and Norwegian Elkhound dog breeds [17, 18]. Functional work would be required to further understand the roles of the ADAMTS family in glaucoma disease pathogenesis.

In summary, we have used a novel GWAS approach and genome sequencing to identify an inversion associated with POAG in the PBGV dog breed. The approach further highlights how the increasing availability and cost effectiveness of massively parallel sequencing are facilitating studies into inherited disease in the dog.

## Materials and Methods

### Ethics Statement

Collection of DNA samples was performed by buccal swabbing, which is a non-invasive technique that does not require a United Kingdom Home Office License. Only pet dogs were used in the study with full owner consent. Tissue samples for the study into primary open angle glaucoma were obtained after enucleation of a glaucoma case by a veterinary ophthalmologist on welfare grounds due to the severity of clinical signs (carried out in accordance with the Veterinary Surgeons Act 1966 and under the auspices of the RCVS). Full owner consent was obtained. As the techniques used were either non-invasive or in the case of eye surgery, were required to alleviate animal suffering rather than for research purposes, no ethics committee approval was required.

### Diagnosis of POAG cases, and sample set selection

All cases of POAG were diagnosed by a specialist veterinary ophthalmologist. The basic clinical examination for POAG involves the use of slit lamp biomicroscopy, both indirect and direct ophthalmoscopy, applanation tonometry and gonioscopy. Tonometry and gonioscopy are completed before inducing mydriasis using 1% tropicamide, slit lamp biomicroscopy utilised pre and post mydriasis and ophthalmoscopy post mydriasis. Vision assessment is based on history, the menace and dazzle reflexes and, where possible, maze performance.

### DNA extraction and genotyping

DNA was extracted from buccal swabs using the QIAamp Midi kit (Qiagen). Genotyping of the POAG associated inversion was performed by analysis of a fragment length polymorphism generated by PCR. Primers for PCR were as follows: POAG_F, 6FAM-AGGCTCAGAGGAGGGTGACT; POAG_R1, ACAAGGACAAAGCTGTCTGTGA; POAG_R2, ACACAAAGCACCCATGACAG. PCRs were carried out in 12 ul volumes consisting of 1.5 mM dNTPs, 1x Qiagen PCR buffer, 0.5 μM of each primer, 0.6U of Qiagen HotStarTaq polymerase and template DNA. Thermal cycling consisted of 5 minutes at 95°C, followed by 35 cycles of 95°C for 30 seconds, 57°C for 30 seconds, and 72°C for 30 seconds, with a final elongation stage of 72°C for 5 minutes. Products of PCR were analysed using the fragment analysis module of an ABI3130xl genetic analyser. Sequencing of the *ADAMTS17* gene was carried out as previously described [11].

### Exome sequencing and allelic association analysis

Libraries for exome sequencing of 12 POAG cases and 12 breed matched controls were made using the Illumina Nextera Exome Enrichment kit according to the manufacturer’s instructions. Libraries were quantified by qPCR using the KAPA library quantification kit. Sequencing of libraries was performed using two runs on the Illumina MiSeq platform, generating paired-end reads of 250 bp in length. The sequence reads generated were aligned to the canine reference genome build, CanFam3.1 using BWA [19]. Variants were called using the GATK [20]. Variants were converted to PLINK format for use in allelic association analysis using VCFtools [21]. Allelic association analyses were carried out using the whole genome data analysis toolset, PLINK [22]. Genome sequencing was outsourced to the Wellcome Trust Centre for Human Genetics, Oxford. Sequencing data can be found in the European Nucleotide Archive, study accession number PRJEB11835.

### Expression analysis

RNA was extracted from retina using the Qiagen RNeasy Midi kit, and included an on column DNase treatment. Isolation of mRNA from total RNA was performed using Sera-Mag oligo-dT beads. Libraries for RNAseq were generated using NEBNext Ultra RNA Library Prep Kit for Illumina sequencing. Sequencing was performed on an Illumina MiSeq generating a dataset of 75 bp paired-end reads, using one run per RNAseq library. Approximate dataset sizes were 4 Gb.

Synthesis of cDNA for qPCR expression analysis was performed using the Qiagen Quantitech reverse transcription kit. Expression analysis by qPCR was performed in 8 μl reactions, containing 1x primer- probe mix, 1x KAPA Probe Fast qPCR Master Mix and 2 μl template cDNA. Standard curves were generated over a seven point, two fold dilution. Standard curves for all qPCR assays had an r^2^ of greater than 0.99 and all assays had an efficiency of greater than 95%. Primers for qPCR, reaction efficiencies and full datasets with calculations can be found in S1 Dataset.

## Supporting Information

S1 Dataset qRT-PCR primer sequences, reaction efficiencies and calculations. (XLSX)

S1 Fig Association analysis using a Mixed Model approach correcting for population structure for the POAG GWAS. (TIF)

S1 File Sequence information for novel *ADAMTS17* exons. (DOCX)

S1 Table Genes in the disease-associated interval for POAG. (DOCX)

S2 Table Non-synonymous SNP variants in the POAG disease-associated interval. (DOCX)
