# Supplementary figures and images for: A Novel Genome-Wide Association Study Approach Using Genotyping by Exome Sequencing Leads to the Identification of a Primary Open Angle Glaucoma Associated Inversion Disrupting ADAMTS17
Source: PLoS One. 2015 Dec 18;10(12):e0143546. doi: 10.1371/journal.pone.0143546 (PMC4684296; doi:10.1371/journal.pone.0143546)

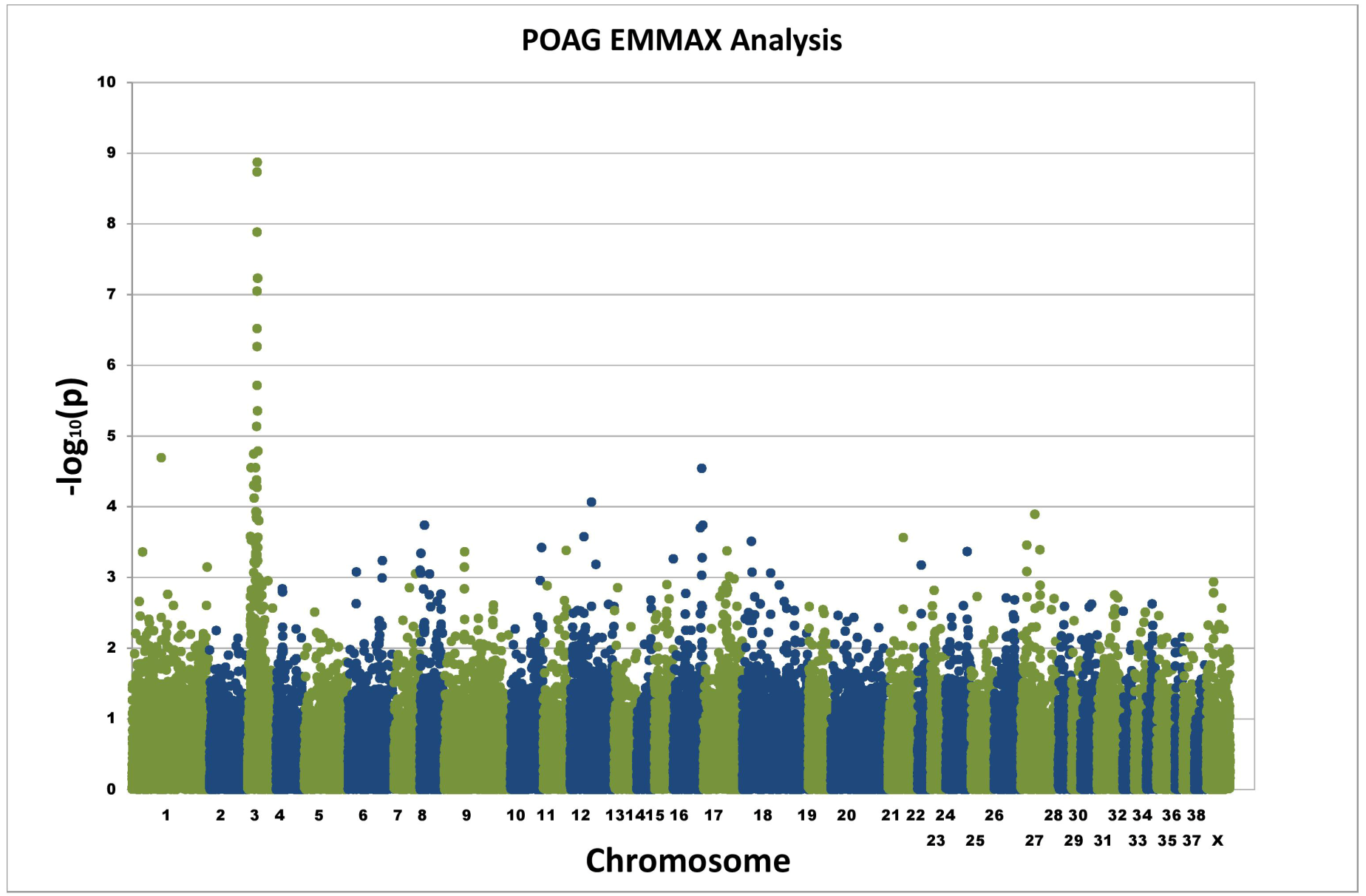

Supplement: S1 Fig — (TIF) [file pone.0143546.s002.tif]
