# Supplementary material for: A Novel Genome-Wide Association Study Approach Using Genotyping by Exome Sequencing Leads to the Identification of a Primary Open Angle Glaucoma Associated Inversion Disrupting ADAMTS17
Source: PLoS One. 2015 Dec 18;10(12):e0143546. doi: 10.1371/journal.pone.0143546 (PMC4684296; doi:10.1371/journal.pone.0143546)
Supplement: S1 File — (DOCX) [file pone.0143546.s003.docx]

**Further information on novel *ADAMTS17* exons**

**chr3:45,701,021-45,702,755 (-strand)**

Highlighted bases are from exon 12 (i.e. the exon before the inversion)

CCGAGAGAGTTA**TAA**ACATGGCATCTCCAGAAGAGAAGGCAGCTATGGAGGTTGGGACAAACTATTCAAAATGGAAATAGTTAACAGAAATCTTGATGGAGAACGACACCGTCTCTGCATCTGTTTGTGAACGTGCAGGGCAATGTGCTGTGAGGTGGGCGATTTGCACTAACAGGAGTGCTAATATGATGTATTAAATGTCAGACCAGCATTGCTGGGTGTGTGTATCTCTCCTGTGGTACTGCCTGTTTATGCTTATTCAGCCCTTTGAATAATTTAACACATTTCATACTCTAAATTACTCTCAGCAATTTGATGGGGCTTCTGGCAGTTTACTTGAAACGCTCAGTGTTAGGTCAACATAGGGTCCCAGCTTCTGCTAAATAGAAGTACTCTGCCTACTTAGGAAAGCATAATTAGTTTTCTCCATGGATCTCCTGGAGGTTGGGGTGAGGTGGAAGAATAGGTGGGGGGAGCCAGGAGGTAGACACTGCATGTGTTCATTCGATTGTCAGTCCAGAAAATGCCAAGGATCCAAAGGTTAAAAATTTAATGGGGCAGGAAGCTGGCCTTCAGGAGAAGGAAAAGTTGAAGGGCATGGGAAAACAGTGTGAGTGTGCTGTGCAAATGGGTGTGCATGATAAATTACCCTCATTTAGTCTATCTTGACAGCAGCTGGAGTCCCTTTTCCATTTAATCCTGATGTAAAAGTGGCCTGTCCCAGTGAAGACTGGCCACAGTGTGTGGAGAAAGTGCTATAGGGTGCAGTAATTTGTGCAAGTGAAACAGTGCACCTCTATCTGATCAGGAGCCTCTTCATCACTGCTATTTCCAGTTTTCGATATAATCTGCAGCGATGATGCAAAATTCCTGAAAATCTTATTTTTTCCTTCTCCCCATGGAACCACTATTTCTATGCATATAAAGAATTAAACTTGGCTCAGAGCTGGAGGGACACCAGTTTCTTTAATATCCTAAGAGTGTGGTTTTAAGAAGTATCTTTCCCTTCACCCTTTTCTCCCTCTCTCTTCTGTGACATCCAGGATGGAAAGGGCATGGACCTTATGAGAGGTGAGCATTTTCAATTAGGACTTAAAATCTTACCTTTGATGATGCATCACCTTGAGTGAAATCCCAAGTATTCTAATGACCTCGGCCAGCCAATGTGTCAGCAAAATGATGATGCAAGAGACCAGGGGCTCAGCTCTTTGTTATGTGACAGCAGGACTCTGATTGACTGTACCCTCTGGCTTTGAACTCCTCCACCACTCTCTGGATATGCTGAAAAATCTTCTTGGGGATTTGAACAGTCTTACACATACTGAGACTCCAGAATGAAGTGTTTGCTTATCATCCATTTTTACCCCTTTTGAAATAGTCTGAGCTTGCATTGTAACCTTAGGTCAAAACTATTTGGGGTGGTGGAAACTCATGATAAACACATGCTTTTGAAGGTTAGAATGCCATCAGTTTAAGAACATCAGATTAACCTTTTTATTTAAAATTTTAAAAAAAAAACACTCCAAAGTGGTAGGAAGACCACATGTGTATCTCTACCTACATGCATGCATTAAAAATACTTGGAGACAAAAAGAGAAATGTTTGCACACCAAAATATATAGTCATCTATTAAAACTATTTTGGTTAAGAAATTAATGATATTTTGAACAACTAGCAGTCTTTTAAAAAATCTGTGTATTTGTCATCCTTTGCTATAAAAAAAAGAGACATTCCATC

5'3' Frame 1

PREL-

**chr3:45,716,674-45,716,889 (-strand)**

Insufficient data to calculate splice junction position. All possible frames include stop codons.

CTTGGATTAGAACCACTTCTACATTGATTAGGACCACTTCAAGTACAGGAAAGGGAAATGTATCTTTGAATAGCCCATCTTCAAAATGCCAGTAATCCAAGAAAGTAA

5'3' Frame 1

LGLEPLLH-LGPLQVQEREMYL-IAHLQNASNPRK-

5'3' Frame 2

LD-NHFYID-DHFKYRKGKCIFE-PIFKMPVIQES

5'3' Frame 3

WIRTTSTLIRTTSSTGKGNVSLNSPSSKCQ-SKKV
