# Supplementary material for: A Novel Genome-Wide Association Study Approach Using Genotyping by Exome Sequencing Leads to the Identification of a Primary Open Angle Glaucoma Associated Inversion Disrupting ADAMTS17
Source: PLoS One. 2015 Dec 18;10(12):e0143546. doi: 10.1371/journal.pone.0143546 (PMC4684296; doi:10.1371/journal.pone.0143546)
Supplement: S1 Table — (DOCX) [file pone.0143546.s004.docx]

Supplementary File S2

| Chromosome | **Gene Name** | **Gene Start (bp)** | **Gene End (bp)** | **Ensembl Gene ID** | **Descriptive Name** |
| --- | --- | --- | --- | --- | --- |
| 3 | Novel | 44624606 | 44624743 | ENSCAFG00000026734 | Novel |
| 3 | Y_RNA | 44088224 | 44088320 | ENSCAFG00000021411 | Y RNA |
| 3 | U4 | 46723899 | 46724061 | ENSCAFG00000028217 | U4 spliceosomal RNA |
| 3 | U6 | 44022278 | 44022383 | ENSCAFG00000027621 | U6 spliceosomal RNA |
| 3 | Novel | 44141085 | 44141130 | ENSCAFG00000027066 | Novel |
| 3 | SNORA31 | 44930099 | 44930197 | ENSCAFG00000027777 | Small nucleolar RNA SNORA31 |
| 3 | Uncharacterized | 45309826 | 45310404 | ENSCAFG00000029940 | Uncharacterized protein |
| 3 | Uncharacterized | 47044075 | 47044950 | ENSCAFG00000029873 | Uncharacterized protein |
| 3 | Novel | 40225528 | 40226554 | ENSCAFG00000013279 | Novel |
| 3 | ASB7 | 40365355 | 40414671 | ENSCAFG00000010679 | ankyrin repeat and SOCS box containing 7 |
| 3 | LINS | 40416506 | 40438345 | ENSCAFG00000010684 | lines homolog (Drosophila) |
| 3 | CERS3 | 40448108 | 40570828 | ENSCAFG00000010691 | ceramide synthase 3 |
| 3 | **ADAMTS17** | **40613857** | **40939685** | **ENSCAFG00000010709** | **ADAM metallopeptidase with thrombospondin type 1 motif, 17** |
| 3 | LYSMD4 | 40983367 | 40989798 | ENSCAFG00000028942 | LysM, putative peptidoglycan-binding, domain containing 4 |
| 3 | MEF2A | 41015815 | 41108399 | ENSCAFG00000010775 | myocyte enhancer factor 2A |
| 3 | LRRC28 | 41332829 | 41492580 | ENSCAFG00000010811 | leucine rich repeat containing 28 |
| 3 | TTC23 | 41505755 | 41592737 | ENSCAFG00000010827 | tetratricopeptide repeat domain 23 |
| 3 | SYNM | 41597899 | 41623480 | ENSCAFG00000010852 | synemin, intermediate filament protein |
| 3 | IGF1R | 41794629 | 42090208 | ENSCAFG00000010881 | Tyrosine-protein kinase receptor |
| 3 | PGPEP1L | 42101083 | 42146818 | ENSCAFG00000010891 | pyroglutamyl-peptidase I-like |
| 3 | FAM169B | 42154880 | 42239983 | ENSCAFG00000010895 | family with sequence similarity 169, member B |
| 3 | ARRDC4 | 42650202 | 42664423 | ENSCAFG00000010896 | arrestin domain containing 4 |
| 3 | Novel | 44084153 | 44142321 | ENSCAFG00000010911 | Novel |
| 3 | Novel | 44084153 | 44142321 | ENSCAFG00000010911 | Novel |
| 3 | MCTP2 | 45845658 | 46036742 | ENSCAFG00000010940 | multiple C2 domains, transmembrane 2 |
| 3 | RGMA | 47044073 | 47088294 | ENSCAFG00000010973 | repulsive guidance molecule family member a |
| 3 | CHD2 | 47101723 | 47220485 | ENSCAFG00000010998 | chromodomain helicase DNA binding protein 2 |
| 3 | ALDH1A3 | 40130898 | 40168752 | ENSCAFG00000010617 | aldehyde dehydrogenase 1 family, member A3 |
