# Supplementary material for: A Novel Genome-Wide Association Study Approach Using Genotyping by Exome Sequencing Leads to the Identification of a Primary Open Angle Glaucoma Associated Inversion Disrupting ADAMTS17
Source: PLoS One. 2015 Dec 18;10(12):e0143546. doi: 10.1371/journal.pone.0143546 (PMC4684296; doi:10.1371/journal.pone.0143546)
Supplement: S2 Table — (DOCX) [file pone.0143546.s005.docx]

Supplementary File S3

| **Location** | **Gene** | **Ref** | **Allele** | **Consequence** | **CDS_position** | **Codons** | **Protein_position** | **Amino_acids** | **Existing_variation** | **SIFT** |
| --- | --- | --- | --- | --- | --- | --- | --- | --- | --- | --- |
| 3:40037706 | LRRK1 | A | C | missense_variant | 2460 | caT/caG | 820 | H/Q | - | tolerated(0.35) |
| 3:40037706 | LRRK1 | A | C | missense_variant | 2436 | caT/caG | 812 | H/Q | - | tolerated(0.35) |
| 3:40168587 | ALDH1A3 | A | C | missense_variant | 17 | tTg/tGg | 6 | L/W | - | deleterious(0.02) |
| 3:40168588 | ALDH1A3 | A | C | missense_variant | 16 | Ttg/Gtg | 6 | L/V | - | tolerated(0.16) |
| 3:40429653 | LINS | T | G | missense_variant | 281 | tTc/tGc | 94 | F/C | - | tolerated(0.18) |
| 3:40436301 | LINS | T | G | missense_variant | 1636 | Tgg/Ggg | 546 | W/G | - | deleterious(0) |
| 3:40987189 | LYSMD4 | A | T | missense_variant | 386 | gAt/gTt | 129 | D/V | - | deleterious(0) |
| 3:41598215 | SYNM | G | A | missense_variant | 4253 | gCg/gTg | 1418 | A/V | - | tolerated(0.11) |
| 3:41599598 | SYNM | C | T | missense_variant | 2941 | Ggc/Agc | 981 | G/S | - | tolerated(0.09) |
| 3:41623034 | SYNM | G | C | missense_variant | 361 | Cag/Gag | 121 | Q/E | - | tolerated(0.25) |
| 3:41623150 | SYNM | G | T | missense_variant | 245 | gCg/gAg | 82 | A/E | rs23602445 | tolerated(1) |
| 3:42663358 | ARRDC4 | A | C | missense_variant | 154 | Ttg/Gtg | 52 | L/V | - | tolerated(1) |
